# Targeting suicidal ideation in major depressive disorder with MRI-navigated Stanford accelerated intelligent neuromodulation therapy

**DOI:** 10.1038/s41398-023-02707-9

**Published:** 2024-01-10

**Authors:** Baojuan Li, Na Zhao, Nailong Tang, Karl J. Friston, Wensheng Zhai, Di Wu, Junchang Liu, Yihuan Chen, Yan Min, Yuting Qiao, Wenming Liu, Wanqing Shu, Min Liu, Ping Zhou, Li Guo, Shun Qi, Long-Biao Cui, Huaning Wang

**Affiliations:** 1 School of Biomedical Engineering, Fourth Military Medical University, 710032 Xi’an, Shaanxi China; 2 Center for Cognition and Brain Disorders, the Affiliated Hospital of Hangzhou Normal University, 310015 Hangzhou, Zhejiang China; 3 Institute of Psychological Sciences, Hangzhou Normal University, 311121 Hangzhou, Zhejiang China; 4 Zhejiang Key Laboratory for Research in Assessment of Cognitive Impairments, 310015 Hangzhou, Zhejiang China; 5 Department of Psychiatry, Xijing Hospital, Fourth Military Medical University, 710032 Xi’an, China; 6 Department of Psychiatry, 907 Hospital of Joint Logistics Team, 353000 Nanping, Fujian China; 7 The Wellcome Centre for Human Neuroimaging, UCL Queen Square Institute of Neurology, London, WC1N 3AR UK; 8 Brain Modulation and Scientific Research Center, 710043 Xi’an, China; 9 Neuromodulation Lab of Brain Science and Humanoid Intelligence Research Center, Xi’an Jiaotong University, 710049 Xi’an, China; 10 Shaanxi Provincial Key Laboratory of Clinic Genetics, Fourth Military Medical University, 710032 Xi’an, China; 11 Department of Radiology, The Second Medical Center, Chinese PLA General Hospital, 100856 Beijing, China

**Keywords:** Depression, Neuroscience

## Abstract

High suicide risk represents a serious problem in patients with major depressive disorder (MDD), yet treatment options that could safely and rapidly ameliorate suicidal ideation remain elusive. Here, we tested the feasibility and preliminary efficacy of the Stanford Accelerated Intelligent Neuromodulation Therapy (SAINT) in reducing suicidal ideation in patients with MDD. Thirty-two MDD patients with moderate to severe suicidal ideation participated in the current study. Suicidal ideation and depression symptoms were assessed before and after 5 days of open-label SAINT. The neural pathways supporting rapid-acting antidepressant and suicide prevention effects were identified with dynamic causal modelling based on resting-state functional magnetic resonance imaging. We found that 5 days of SAINT effectively alleviated suicidal ideation in patients with MDD with a high response rate of 65.63%. Moreover, the response rates achieved 78.13% and 90.63% with 2 weeks and 4 weeks after SAINT, respectively. In addition, we found that the suicide prevention effects of SAINT were associated with the effective connectivity involving the insula and hippocampus, while the antidepressant effects were related to connections of the subgenual anterior cingulate cortex (sgACC). These results show that SAINT is a rapid-acting and effective way to reduce suicidal ideation. Our findings further suggest that distinct neural mechanisms may contribute to the rapid-acting effects on the relief of suicidal ideation and depression, respectively.

## Introduction

Suicide risk in patients with major depressive disorder (MDD) is 20 times more than that of the general population [1, 2], and suicidal behavior exists at all times during major depressive episodes [3]. The lifetime prevalence of suicide attempts in patients with MDD is 31% [4]. and more than half of patients experience suicidal ideation beforehand [5]. Suicide-related costs account for about 5% of the total incremental costs of MDD adults [6], representing a substantial burden to patients and their families.

The general treatments for alleviating suicidal ideation include various antidepressants [7–9], lithium [10, 11], ketamine [12, 13], electric convulsive therapy (ECT) [14, 15], and cognitive behavioral therapy (CBT) [16]. However, the antidepressants/lithium and psychotherapy usually require weeks to exert anti-suicide effects. Furthermore, antidepressants may increase suicide risk in children and adolescents, as well as adults in early-phase pharmacotherapy [17, 18]. ECT is an effective way to rapidly relieve suicidal ideation, but the tolerability and complex side effects limit its application [19]. Evidence also suggests that ketamine may be a promising rapid-acting option, but its effects seem to be short-lived [12]. The problems of current treatments motivate the search for safe and rapid relief interventions for suicidal ideation in patients with MDD.

Recent evidence suggests that a non-invasive treatment option, i.e., repetitive transcranial magnetic stimulation (rTMS), may be a rapid and safe way in relieving both depression and suicidal ideation. As recommended by the “Evidence-based guidelines on the therapeutic use of repetitive transcranial magnetic stimulation (rTMS): An update (2014-2018)”, level A evidence (definite efficacy) is proposed for high-frequency (HF)-rTMS on the left dorsal lateral prefrontal cortex (DLPFC) in MDD [20]. Actually, MRI-navigated rTMS has shown high efficacy and rapid action in the treatment of depression. Recently, an individualized accelerated, high-dose intermittent theta-burst stimulation (iTBS) protocol, i.e., Stanford Neuromodulation Therapy (SAINT), was proposed recently by Williams et al. [21]. The safety, effectiveness, and rapid action of this protocol have been validated with both open-label and double-blind studies. Treatment with five days has shown a high response rate of 85.7% and remission rate of 78.6% for treatment-resistant depression [22]. SAINT has been approved by FDA as an effective way for the treatment of refractory depression. Notably, it has also been shown to be a potential way to rapidly reduce the severity of suicidal ideation [23].

Although SAINT appears to be highly effective, the neural mechanisms underwriting its rapid-acting antidepressant and suicide prevention effects remain unclear. The brain is a complex network comprising functionally specialized regions that flexibly interact to support a diverse repertoire of cognitive and behavioral functions [24, 25]. Characterizing the brain’s connectivity, which constitutes a functional connectome “fingerprint” [26], may help to elucidate the neural mechanisms supporting the rapid-acting effects of SAINT. Indeed, accumulating evidence shows that the therapeutic efficacy of rTMS might be closely associated with the functional connectivity of its stimulation target on the DLPFC with, the subgenual anterior cingulate cortex (sgACC) [27–31]. However, the modulatory effects of rTMS are not only restricted to the DLPFC-sgACC connectivity, but also manifest in distributed brain networks associated with depression, such as the default mode network (DMN) [32, 33], affective network (AN) [34], salience network (SN) [35], reward network (RN) [36], and visual network (VN) [35]. How does the therapeutic intervention transmit from the stimulation target to distributed networks? Could the neural pathways conveying rTMS stimulation account for its rapid-acting antidepressant and suicide prevention effects?

To address above-mentioned issues, we collected functional magnetic resonance imaging (fMRI) images in 32 MDD patients with suicidal ideation before and immediately after 5-day SAINT. We investigated the information flow from the rTMS target to core regions associated with depression and suicide ideation using effective connectivity analysis based on dynamic causal modelling (DCM). Effective connectivity differs from conventional functional connectivity simply computing the correlation among time courses of interacting regions. Instead, it could infer the causal influences from one region to another and depict the signal flow directions within a brain network. Our results showed that the rapid-acting antidepressant effects of SAINT were related to effective connections of the sgACC, while the suicide prevention effects were more associated with the effective connectivity of the insula (INS).

## Methods

### Participants

The study was approved by the Ethics Committee of the First Affiliated Hospital, Fourth Military Medical University, and was conducted in accordance with the Declaration of Helsinki (clinicaltrial.gov identifier: NCT04653337). Written informed consents were obtained from all the participants.

All patients were recruited from the Department of Psychiatry at the First Affiliated Hospital, Fourth Military Medical University, from January 2021 to October 2021, according to the following criteria: (i) 18–60 years old; (ii) meeting the criteria of the Diagnostic and Statistical Manual of Mental Disorder, Fifth Edition (DSM-5) for patients with unipolar MDD assessed by Mini-Neuropsychiatric Interview (MINI); (iii) right handedness; (iv) with a score > 17 on the 17-item Hamilton Depression Rating Scale (HAMD-17) [37]; (v) with a score ≥ 6 on the Beck Scale for Suicidal ideation-Chinese Version (BSI-CV) [5, 38]; (vi) normal results on physical examination and electroencephalography. We excluded those patients with (i) received antidepressant treatment 2 months prior to the study; (ii) any other current or past psychiatric axis-I or axis-II disorders; (iii) severe physical illnesses; (iv) psychotic symptoms, alcohol or drug abuse; (v) a history of neurological disorders including seizure, cerebral trauma, or MRI evidence of structural brain abnormalities; (vi) contraindications to MRI and rTMS, such as metallic implants in the body, cardiac pacemakers, claustrophobia, etc.; (vii) acute suicide or self-injury behavior in need of immediate intervention; (viii) pregnancy, lactation, or a planned pregnancy for females.

**Fig. 1:**
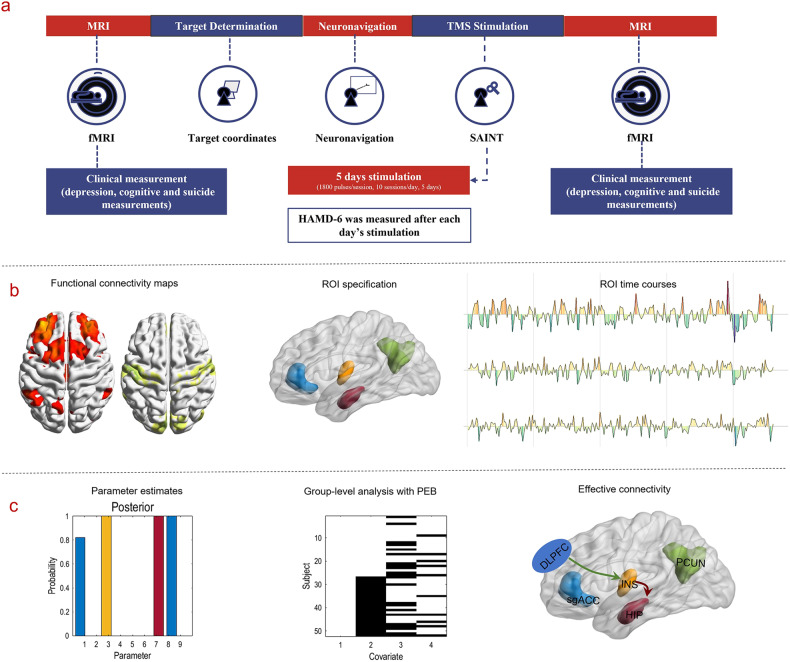
Workflow for the whole study. **a** Flowchart of the trial. **b** Target-based functional connectivity profiles and ROIs selection for the following effective connectivity analysis. **c** Illustrations of dynamic causal modeling for effective connectivity analysis.

Thirty-four participants were enrolled in this study. Two patients withdrew from the study due to personal reasons after the first day of treatment. For ethical and safety reasons, venlafaxine (75 mg/d) or duloxetine (30 mg/d) were prescribed at the beginning of the treatment. Dexzopiclone or zolpidem was also used to improve the sleep quality of individuals who suffered from severe insomnia. Figure 1 describes the workflow of the study, and the demographic characteristics of the patients are provided in the supplementary Table 1.

### Clinical assessments

Suicidal ideation and depression symptoms were assessed by clinical and self-report scales at baseline, immediately after SAINT (after the last session of SAINT), 2 and 4 weeks after the whole SAINT. The severity of suicidal ideation was measured by BSI-CV, item 3 of the HAMD-17, and item 10 of the Montgomery-Asberg Depression Rating Scale (MADRS). Depression symptoms were assessed with HAMD-17 and MADRS. At the end of each day’s treatment, 6-item HAMD (HAMD-6) was also used to assess the depression symptoms. Potential neurocognitive side effects were assessed using a neuropsychological test battery before and immediately after SAINT, including Perceived Deficits Questionnaire-Depression (PDQ-D) [39], Digital Span Test (DST) [40], and Digit Symbol Substitution Test (DSST) [41].

**Fig. 2:**
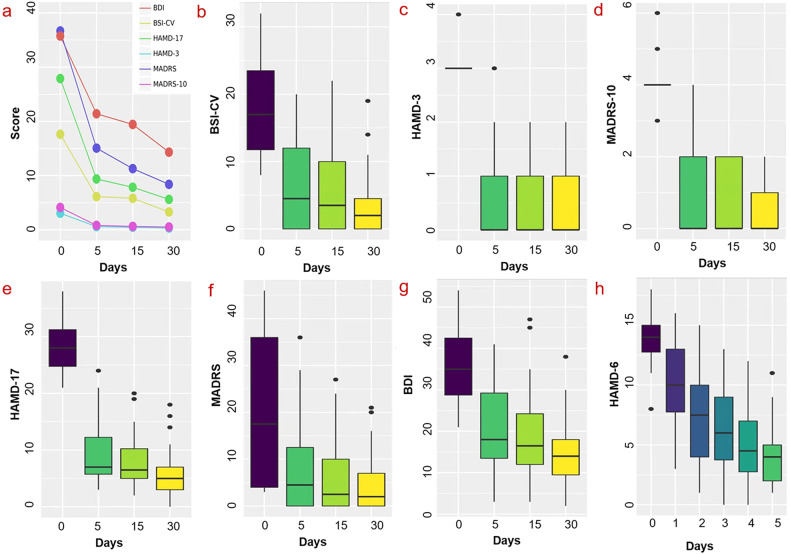
Changes in scale score during and after SAINT in MDD patients with suicidal ideation. **a**, **b** Significant decrease in the BSI-CV (*F* = 81.34, *df* = 2, 61, *p* < 0.001). **a**, **c** Significant decrease in item 3 of the HAMD-17 (*F* = 317.90, *df* = 2, 66, *p* < 0.001). **a**, **d** Significant decrease in item 10 of the MADRS (*F* = 314.72, *df* = 2, 64, *p* < 0.001). **a**, **e** Significant decrease in HAMD-17 (*F* = 267.30, *df* = 3, 93, *p* < 0.001). **a**, **f** Significant decrease in MADRS (*F* = 351.73, *df* = 2, 68, *p* < 0.001). **a**, **g** Significant decrease in BDI (*F* = 67.99, *df* = 3, 93, *p* < 0.001). **a**, **h** A significant effect of day on mean HAMD-6 scores (*F* = 102.67, *df* = 3, 95, *p* < 0.001). ***p < 0.001; BSI-CV: Chinese Version of the Beck Scale for Suicidal Ideation. HAMD-3: the 3rd item of HAMD-17; MADRS-10: the 10th item of Montgomery–Asberg Depression Rating Scale; HAMD-17: 17-item HAMD; MADRS: Montgomery–Asberg Depression Rating Scale; BDI: Beck Depression Inventory; HAMD-6: 6-item Hamilton Depression Rating Scale.

BSI-CV scores were the main (clinical) outcomes of the study. The suicidal ideation response was defined as a reduction ≥ 50% on the BSI-CV, while the remission of suicidal ideation was defined as a reduction ≥ 50% and < 6 on the BSI-CV. Response to depression symptoms was defined as a reduction ≥ 50% on the HAMD-17, MADRS, and HAMD-6 scales. The remission of depression symptoms was defined as a score < 8 on the HAMD-17 [42], a score < 11 on the MADRS [43], a score < 5 on the HAMD-6 [44], and a score < 13 on the BDI [45]. All statistical analyses of clinical data were conducted using SPSS, version 26 (IBM, Armonk, N.Y.). The level of statistical significance was set at *p* = 0.05. As one patient failed to participate the clinical assessment 4 weeks after SAINT, the mean value of all participants’ clinical score at that time point was used to replace missing data. Changes in BSI-CV, HAMD-17, HAMD-6, MADRS scores were assessed with repeated measures ANOVA, while changes in PDQ-D, DST, DSST were evaluated with paired *t* tests. The relevant results are displayed in Table 1 and Fig. 2.

**Table 1:** Clinical measurements of the patients at baseline and follow up.

Measurement	Baseline		Post-SAINT				2 weeks after SAINT				4 weeks after SAINT			
	Mean	SD	Mean	SD	Response No. (%)	Remission No. (%)	Mean	SD	Response No. (%)	Remission No. (%)	Mean	SD	Response No. (%)	Remission No. (%)
*Suicidal ideation*														
BSI-CV	17.63	7.06	6.13	5.98	21 (65.63)	18 (56.25)	5.81	6.01	25 (78.13)	19 (59.38)	3.39	4.53	29 (90.63)	24 (75.00)
HAMD, item 3	3.00	0.00	0.30	0.50	–	–	0.30	0.50	–	–	0.20	0.40	–	–
MADRS, item 10	3.90	0.30	0.50	0.80	–	–	0.50	0.80	–	–	0.30	0.70	–	–
*Depression symptoms*														
HAMD-17	27.91	4.31	9.36	5.43	26 (81.25)	17 (53.13)	7.84	4.57	29 (90.63)	18 (56.25)	5.72	4.24	30 (93.75)	26 (81.25)
MADRS	36.69	4.49	15.06	7.24	25 (78.13)	9 (28.13)	11.28	5.67	29 (90.63)	18 (56.25)	8.45	5.17	31 (96.88)	25 (78.13)
BDI	35.75	9.17	21.41	10.92	13 (40.63)	8 (25.00)	19.44	10.33	20 (62.50)	10 (31.25)	14.33	7.75	23 (71.88)	15 (46.88)
HAMD-6	13.63	2.17	4.41	2.66	26 (81.25)	19 (59.38)	–		–	–	–		–	–
*Neurocognitive test*														
PDQ-D	42.13	15.65	30.22	17.27	–	–	–		–	–	–		–	–
DST	13.50	2.31	14.88	2.54	–	–	–		–	–	–		–	–
DSST	55.19	11.60	63.63	11.89	–	–	–		–	–	–			

*N* the number of participants, *mean* the mean value of measurement scores, *SD* standard deviation, *HAMD-6* 6 item Hamilton Depression Rating Scale (6-item HAMD), *HAMD-17* 17 item HAMD, *MADRS* Montgomery–Asberg Depression Rating Scale, *BDI* Beck Depression Inventory, *BSI-CV* Chinese Version of the Beck Scale for Suicidal Ideation, *PDQ-D* Perceived Deficits Questionnaire-Depression, *DST* Digital Span Test, *DSST* Digit-symbol Substitution Test.

### Procedures of MRI-navigated rTMS

The MRI-navigated rTMS treatment was delivered by a Black Dolphin Navigation Robot system (SmarPhin S-50, Solide Brain Control Medical Technology Co., Ltd., Xi’an, China). The individualized rTMS stimulation target is defined as the peak subunit on the DLPFC that was mostly negatively connected to the sgACC according to Cole et al. [23]. Whereas the definition of the sgACC was slightly different from that of Cole et al. [23]. In the current study, No. 187 and 188 atlases based on Brainnetome Atlas (BNA) (https://atlas.brainnetome.org/bnatlas.html) [46] were selected as the sgACC to improve the signal noise ratio and avoid mixing information comes from the corpus callosum. After the definition of the individualized stimulation target, 5-day sgACC FC-guided rTMS treatment, i.e., SAINT, was given for each patient [22, 23]. Specifically, three consecutive iTBS were delivered at 90% of the resting motor threshold (RMT) for each session in 9 min 52 s. Ten sessions of iTBS (18,000 pulses), with a 50-min interval of each session, were delivered to the subject every day. The whole treatment lasted for 5 consecutive days and 90,000 pulses in total were received by each patient.

### Image acquisition

High-resolution MRI data were acquired on a 3.0 T UNITED 770 scanner before and after treatment. Parameters for 3D-T1-weighted structural imaging were: slices = 192, repetition time = 7.24 ms, echo time = 3.10 ms, slice thickness = 1.0 mm, matrix size= 512 × 512, field of view = 256 × 256 mm^2^, flip angle = 10°. Parameters for resting-state fMRI with eye-closed were: slices =35, repetition time = 2000 ms, echo time = 30 ms, slice thickness = 4 mm, matrix size = 64 × 64, field of view = 224 × 224 mm^2^, flip angle = 90°. The pre-treatment (i.e., baseline) and post-treatment resting-state fMRI sessions lasted for about 12 minutes.

### Data preprocessing

The MRI data were preprocessed with the statistical parametric mapping software package (SPM12, http://www.fil.ion.ucl.ac.uk/spm/software/spm12/) and the GRETNA toolbox (https://www.nitrc.org/projects/gretna/). After discarding the first 10 images due to magnetic field instability, slice timing correction was performed to correct differences in the acquisition time of slices within a volume. Next, realignment was used to correct head motion, and two subjects with translation larger than 3.5 mm or rotation larger than 3.5° were excluded. The images were then normalized to standard Montreal Neurological Institute (MNI) space and spatially smoothed with a Gaussian kernel filter (full width at half maximum (FWHM) = 6 mm). Temporal detrending was used to deal with low-frequency signal drift. Covariates including Friston-24 head motion parameters, signals from white matter, and CSF, were then regressed out. Furthermore, we performed global signal regression to remove spatially coherent confounds [27, 29, 47]. Finally, the fMRI time series were temporally filtered with a bandpass filtering (0.01–0.1 Hz) for functional connectivity analyses. Functional connectivity (i.e., correlation) analyses were used to identify regions of interest (ROIs) for subsequent effective connectivity (i.e., DCM) modelling.

It should be noted that only 28 of the 32 patients completed both the pretreatment and posttreatment MRI scanning. Among them, as 2 patients were excluded because of large head motion, 26 subjects were finally entered into the subsequent fMRI analyses.

### Functional connectivity profiles of the stimulation target

**Fig. 3:**
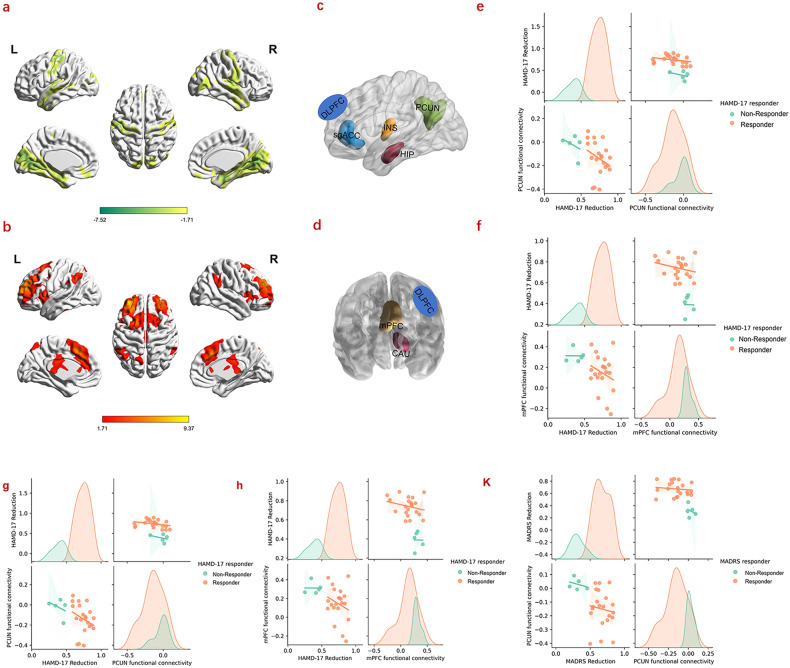
Target-based functional connectivity profiles and the correlations between functional connectivity and clinical scores. **a** Positive functional connectivity profiles of stimulation target. **b** Negative functional connectivity profiles of stimulation target. **c** Selected negative connectivity ROIs based on BNA templates. **d** Selected positive functional connectivity ROIs based on BNA templates. **e**, **f** Correlations between the baseline functional connectivity of the PCUN and mPFC and reductions in HAMD-17 scores. **g**, **h** Correlations between the reductions in HAMD-17 and the functional connectivity of the PCUN (*p* = 0.033) and mPFC (*p* = 0.030) after treatment. **K** Correlation between the reduction in MADRS and the functional connectivity of the PCUN (*p* = 0.015); to directly visualize the differences of the correlations between responders and non-responders, the correlation distributions of responders and non-responders were also plotted, respectively. BNA: Brainnetome Atlas.

Functional connectivity with the individualized stimulation targets was investigated first. In detail, a 6 mm radius spheric ROI centered at the DLPFC target’s MNI coordinates (Supplementary Table 2) for each participant was defined as the seed region. The Pearson correlations between the seed time series and time series of every voxel across the whole brain were then calculated, i.e., target-based functional connectivity (i.e., *r* map). To ensure the Gaussian distribution of residuals of the ensuing parametric tests, all *r* values were Fisher’s Z transformed. One-sample *t*-tests were then conducted on the transformed *z* maps and two signed maps of target-based functional connectivity were obtained, i.e., a positive functional connectivity map and a negative functional connectivity map (Fig. 3). Figure 3a represents regions that negatively correlated with DLPFC targets, while Fig. 3b shows regions that displayed positive correlations with the DLPFC targets (*p* < 0.05, uncorrected).

### Stimulation target-based effective connectivity analysis

Effective connectivity analysis was confined to the left hemisphere regions. Thus, the left caudate (CAU), precuneus (PCUN), hippocampus (HIP), insular (INS) were included in the analysis (Fig. 3c, d). For midline regions, including the midline PFC (mPFC) and sgACC (Fig. 3c, d), the mean time courses of the bilateral cluster were extracted. For each brain region, a binary mask was generated according to the functional connectivity maps and the Brainnetome Atlas (BNA) [46] (https://atlas.brainnetome.org/bnatlas.html) (Supplementary Table 2, Fig. 3a–d). The mean time course of voxels within each mask was then extracted.

Furthermore, to validate whether the depression functional circuit map of our study is consistent with the convergent network proposed by Siddiqi et al. [48], partial correlations between target-based connectivity and depression score changes (including the changes of HAMD-17 and MADRS) with regressing out the baseline depression scores were conducted (Supplementary Fig. 1).

Two DCMs were constructed based on ROIs showing positive and negative correlations with the target ROI. In brief, ROIs from the negative functional connectivity map were combined with the target (seed) ROI to create a fully inter-connected dynamic causal model, named the negative correlation effective connectivity model (NCECM), while the ROIs from the positive functional connectivity map were used to construct the corresponding positive correlation effective connectivity model (PCECM). Directed (i.e., causal) effective connectivity within the NCECM and PCECM were estimated using spectral dynamic causal modeling (spDCM) [49] as follows.

### Effective connectivity analysis with spDCM

The causal interactions among ROIs were modeled with random differential equations for the hidden neuronal states [49]:

$$\dot{x}\left(t\right)=A\,{\cdot }\,x(t)+v(t)$$

Here, *x*(*t*)=[*x*_*1*_(*t*) *x*_*2*_(*t*) *… x*_*n*_(*t*)] ^T^ denotes the hidden neuronal states that represent neuronal activity of the *n* interacting ROIs. *A* represents the effective connectivity characterizing the strength of directed connections among these ROIs, while *v*(*t*) models endogenous fluctuations, with a parameterized spectral profile. The neuronal model is then supplemented with standard hemodynamic state equations that model the translation from unobserved neuronal activity to observed BOLD signals from the ROIs [50]. The model was then inverted, and model parameters were estimated in the frequency domain by fitting complex cross spectra through a Variational Laplace procedure [49]. These (spectral) data features were evaluated prior to the temporal filtering used to identify ultra-slow functional connectivity.

For each subject, a fully connected model with reciprocal connections between all pairs of ROIs was first defined for NCECM and PCECM, respectively. Each fully connected model was then optimized to maximize model evidence (as scored by variational free energy). The posterior probability of the parameters of this fully connected model — from each subject — was then entered into a second-level group analysis. The parametric empirical Bayes (PEB) framework [51, 52] was used to obtain second-level (i.e., between subject and session) commonalities and differences in effective connectivity with a General Linear Model (GLM). The advantage of the PEB framework over classical statistics is that both the posterior expectations and covariance of the parameters are considered when estimating effects at the group level.

In summary, group-level effects were modeled with the following hierarchical model according to [52]:

$${\theta }^{(2)}=\eta +{\varepsilon }^{(3)}$$

$${\theta }^{(1)}=X{\theta }^{(2)}+{\varepsilon }^{(2)}$$

$${Y}_{i}={\varGamma }_{i}\left({{\theta }_{i}}^{(1)}\right)+{X}_{0}{\beta }_{i}+{{\varepsilon }_{i}}^{(1)}$$

Here, *Y*_*i*_ represents the observed BOLD data features of subject *i*. At first level, *Y*_*i*_ is modeled with a DCM ${\varGamma }_{i}$ with parameters ${\theta }_{i}$, a GLM of confounding (and nuisance) effects with design matrix ${X}_{0}$ and parameter ${\beta }_{i}$, and observation noise ${{\varepsilon }_{i}}^{(1)}$. At the second level, DCM parameters ${\theta }_{i}$ are modeled with a second GLM with design matrix *X* and group-level parameters ${\theta }^{(2)}$ which parameterize commonalities and differences in effective connectivity over subjects. The second level GLM included a constant term modelling group means (i.e., commonalities) and differences due to (i) pre-and post- treatment effects, response in terms of (ii) suicidal ideation and (iii) depression (see Fig. 1c—middle panel). ${\varepsilon }^{(2)}$ models random between-subject effects that are not modelled by the GLM. The second-level parameters ${\theta }^{(2)}$ are assumed to have a prior expectation *η* and residuals ${\varepsilon }^{(3)}$. To optimize the ensuing PEB model, Bayesian Model Reduction (BMR) [51] was used to search over all reduced PEB models. Finally, Bayesian Model Averaging (BMA) was employed to summarize connectivity over all plausible (reduced) PEB models.

The ensuing Bayesian model averages of effective connectivity at the second level were used to identify commonalities (i.e., group means) that describe the functional architecture that was conserved over subjects and sessions. The Bayesian model averages of effective connectivity at the first level were used to test for correlations with clinical scores. These Bayesian model averages represent the most efficient estimates of connectivity because they inherit constraints from the second-level GLM.

### Correlations between fMRI connections and clinical scores

To explore whether (functional, and effective) connectivity estimates could predict rTMS treatment effects—in mitigating depression and suicidal ideation symptoms—we calculated the correlations between connectivity estimates and depression (i.e., HAMD-17, MADRS) and suicidal ideation score changes (i.e., BSI-CV), respectively. Furthermore, the correlations between the change percentage in connections and clinical scores (i.e., HAMD-17, MADRS, and BSI-CV) were conducted for exploring the rTMS treatment effect. The post-treatment connectivity estimates were also correlated with clinical scores to explore the after-effect of 5-day treatment.

## Results

For all 26 patients, the MNI coordinates of the stimulation targets, the corresponding superficial depths, resting motor thresholds (RMT) and relevant clinical outcomes were displayed in Table 2. No severe adverse events occurred during the whole trial and the most common side effect was headache (supplementary materials, Supplementary Table 3). All side effects were mild, well tolerated, and resolved rapidly after stimulation.

**Table 2:** MNI coordinates of the stimulation targets for all 26 patients, the corresponding superficial depths, resting motor thresholds (RMT) and relevant clinical outcomes.

Patients	MNI coordinates							
	*x*	*y*	*z*	Superficial Depth (mm)	RMT (%)	BSI-CV (%)	HAMD-17(%)	MADRS (%)
Patient 1	−26	45	28	20.49	65	0.22	0.64	0.54
Patient 2	−26	48	25	22.76	55	1.00	0.76	0.68
Patient 3	−29	49	32	24.86	25	1.00	0.59	0.62
Patient 4	−36	44	34	17.48	70	0.45	0.68	0.63
Patient 5	−27	50	23	22.34	30	1.00	0.79	0.78
Patient 6	−25	53	20	19.11	65	1.00	0.84	0.73
Patient 7	−40	52.5	4	21.53	45	0.72	0.74	0.60
Patient 8	−42	52	13	25.24	55	0.90	0.89	0.75
Patient 9	−32	21	40	21.15	55	0.50	0.48	0.46
Patient 0	−41	53	3	29.46	60	0.84	0.68	0.66
Patient 11	−26	48	27	20.67	60	0.83	0.83	0.83
Patient 12	−31	50	33	24.75	60	0.43	0.78	0.64
Patient 13	−40	50	1	21.92	70	0.36	0.81	0.84
Patient 14	−26	55	28	24.63	70	0.56	0.89	0.69
Patient 15	−30	48	34	17.79	35	0.47	0.86	0.78
Patient 16	−29	45	32	17.93	30	0.88	0.66	0.83
Patient 17	−41	52	8	17.91	60	0.44	0.59	0.26
Patient 18	−26	44	30	22.35	45	0.88	0.71	0.53
Patient 19	−30	48	35	23.17	70	1.00	0.77	0.83
Patient 20	−28	52	25	20.38	45	0.60	0.41	0.33
Patient 21	−28	48	32	20.45	70	0.33	0.69	0.59
Patient 22	−40	53	6	25.88	70	0.39	0.25	0.20
Patient 23	−30	38	30	17.59	75	0.44	0.75	0.50
Patient 24	−27	54	24	20.75	55	0.52	0.46	0.51
Patient 25	−36	33	37	16.78	60	0.33	0.59	0.58
Patient 26	−39	53	−1	17.95	45	0.28	0.34	0.31

*RMT* resting motor threshold, *BSI-CV* Chinese Version of the Beck Scale for Suicidal Ideation, *HAMD-17* 17 item HAMD, *MADRS* Montgomery–Asberg Depression Rating Scale.

### Suicidal ideation

Changes in suicidality scale scores were assessed with a repeated measures ANOVA. After 5 days of treatment, there was significant decrease in the BSI-CV (*F* = 81.34, *df* = 2, 61, *p* < 0.001; Fig. 2a, b), item 3 of the HAMD-17 (*F* = 317.90, *df* = 2, 66, *p* < 0.001; Fig. 2a, c), item 10 of the MADRS (*F* = 314.72, *df* = 2, 64, *p* < 0.001; Fig. 2a, d) at follow-up. The mean BSI-CV score immediately after SAINT reduced by 65.23%. Bonferroni-corrected post-hoc comparisons revealed a significant difference in scores of BSI-CV between 0 and 4 weeks after treatment, while there was no significant difference between 0 and 2 weeks after treatment. Remission and response rates of suicidal ideation after treatment were 56.25% and 65.63% (0 weeks), 59.38% and 81.25% (2 weeks), 75.00% and 93.33% (4 weeks), respectively (Table 1).

### Depression symptoms

Statistical analysis revealed a significant effect of time (weeks) on mean HAMD-17 scores (*F* = 267.30, *df* = 3, 93, *p* < 0.001; Fig. 2a, e) and a significant effect of day on mean HAMD-6 scores (*F* = 102.67, *df* = 3, 95, *p* < 0.001; Fig. 2a, h), with scores at all follow-up time points being significantly lower than at baseline (Bonferroni-corrected pairwise comparisons, *p* < 0.001). These results were recapitulated for the MADRS (*F* = 351.73, *df* = 2, 68, *p* < 0.001; Fig. 2a, f) and the BDI (*F* = 67.99, *df* = 3, 93, *p* < 0.001; Fig. 2a, g). After 5 days of treatment, the mean HAMD-17 score reduced by 66.39%, and the reduction of MADRS was 58.95%. Bonferroni-corrected post-hoc comparisons demonstrated a significant difference in scores of HAMD-17 between 0 and 4 weeks after treatment, while there was no significant difference between 0 and 2 weeks after treatment. The remission rate (HAMD-17 score < 8) and response rate (a reduction ≥ 50% from baseline in HAMD-17) after treatment were 53.13% and 81.25% (0 weeks); 56.25% and 90.63% (2 weeks); 81.25% and 96.88% (4 weeks), respectively (Table 1).

### Functional connectivity with the stimulation target

The stimulation target-based functional connectivity pattern of the pre-treatment is shown in Fig. 3 (a, b; *p* < 0.05 without correction). No significant differences were detected between the pre- and post-treatment (FDR correction, *p* < 0.05).

However, the baseline (pre-treatment) functional connectivity between the DLPFC and PCUN—and between the DLPFC and mPFC—was negatively correlated with the reduction in HAMD-17 (*p* = 0.037, *p* = 0.039) (Fig. 3e, f), respectively. These functional anticorrelations strengthened after rTMS treatment for the PCUN (*p* = 0.033) and for the mPFC (*p* = 0.029), respectively (Fig. 3g, h). Moreover, the MADRS were also negatively correlated with the PCUN connectivity after treatment (*p* = 0.015) (Fig. 3k). We did not find any significant correlations between connectivity and suicidality.

### Stimulation target-based effective connectivity analysis

#### Treatment effect for all subjects

**Fig. 4:**
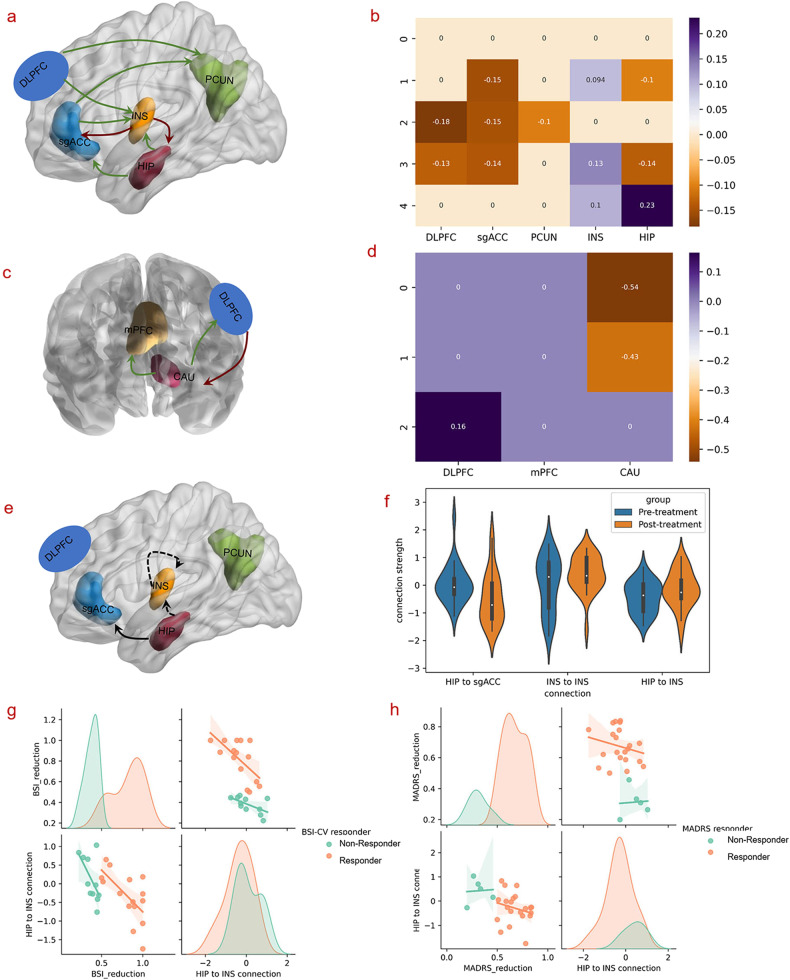
The commonalities and differences of effective connectivity and correlations between effective connectivity and clinical scores; negative value represents inhibitory effects, while positive value indicates excitatory influences. **a**, **b** Commonalities of the effective connectivity between the pre- and pot-treatment in NCECM. **c**, **d** Commonalities of the effective connectivity between the pre- and post-treatment in PCECM. **e**, **f** Significant differences of the effective connectivity between the pre- and post-treatment in NCECM. **g** Anti-correlation between the reduction in BSI-CV scores and the effective connectivity from the HIP to the INS (*p* = 0.001). **h** Anti-correlation between the reduction in MADRS scores and the effective connectivity from the HIP to the INS (*p* = 0.021); same as Fig. 3, to directly visualize the differences of the correlations between responders and non-responders, the correlation distributions of responders and non-responders were also plotted, respectively (**g**, **h**).

In the NCECM (Fig. 4a, b), the stimulation target (DLPFC) exerted inhibitory influences on the PCUN and INS. Signals from the INS are then sent to the sgACC, HIP, and INS itself. Since the connections from DLPFC to INS are inhibitory and connections from the INS to sgACC, INS and HIP are excitatory, these influences together resulted in inhibition and the negative functional connectivity with DLPFC seen in the sgACC, INS and HIP. These inhibitory influences then propagate from the sgACC to PCUN, and from the HIP to sgACC, and INS.

For the PCECM, we only found significant excitatory influences from DLPFC to CAU, followed by inhibitory influences on the DLPFC and mPFC, inhibiting the responses of these two brain regions (Fig. 4c, d).

After 5-day treatment, the self-connection of the INS and the connection from the HIP to INS was significantly increased, while decreases were observed in the connectivity from the HIP to sgACC (Fig. 4e, f). More importantly, the reduction of the BSI-CV scores was negatively correlated with the strength of the connection from the HIP to INS with *p* = 0.001 (Fig. 4g) after rTMS treatment. The MADRS reduction also correlated negatively with the effective connectivity from the HIP to INS (*p* = 0.021) following 5-days of treatment (Fig. 4h).

#### Responders and non-responders to suicidal ideation

**Fig. 5:**
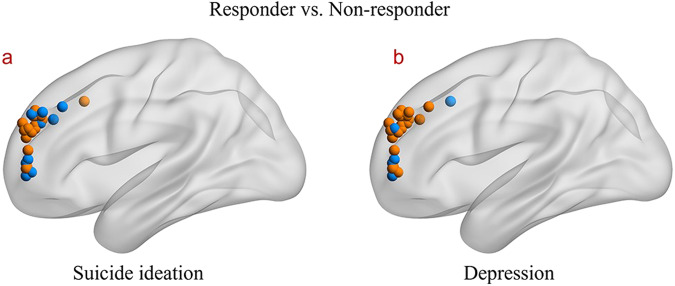
The distribution of the stimulation targets. **a** The distribution of the stimulation targets for responders and non-responders to suicidal ideation. **b** The distribution of the stimulation targets for responders and non-responders to depression. Color orange represents the stimulation targets of responders, while the blue indicates non-responders.

The distributions of the individualized stimulation targets of the responders and non-responders to suicidal ideation were displayed in Fig. 5a.

**Fig. 6:**
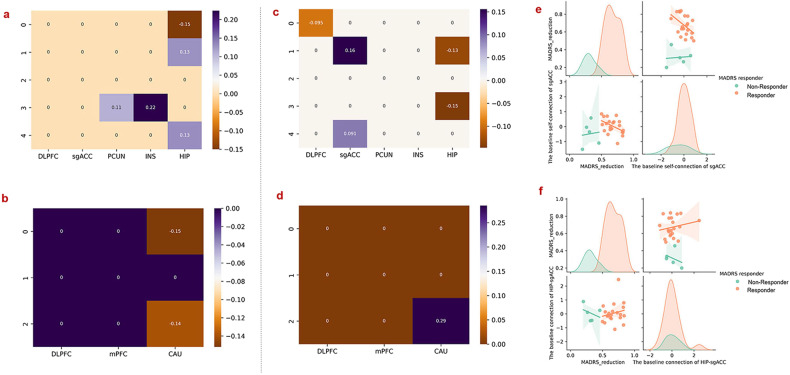
Differences in effective connectivity between responders and non-responders to suicidal ideation and depression symptoms, respectively and the correlations between effective connectivity and clinical scores. **a**, **b** Differences in effective connectivity of the responders and non-responders to suicidal ideation in NCECM and PCECM. **c**, **d** Correlations between the reductions in MADRS score and the effective connectivity after 5-day treatment. **e**, **f** Correlations between the reduction in MADRS scores and the baseline self-connection of the sgACC (*p* = 0.033) and the connection of HIP-sgACC (*p* = 0.040); same as Fig. 3 and Fig. 4, to directly visualize the differences of the correlations between responders and non-responders, the correlation distributions of responders and non-responders were also plotted, respectively (**e**, **f**).

In the NCECM, the responders to suicidal ideation showed significantly increased connectivity from the HIP to DLPFC, whereas connectivity of the PCUN, INS, HIP, and the connection from HIP to sgACC (Fig. 6a) decreased. Meanwhile, differences in connectivity were observed in the connection between the CAU-DLPFC, and CAU self-connection in suicidal ideation responders, compared to non-responders (Fig. 6b).

#### Responders and non-responders to depression

The distributions of individualized stimulation targets of the responders and non-responders to depression were displayed in Fig. 5b.

In contrast to the suicidal ideation pattern, the depression responders showed increased connections in NCECM the from HIP to sgACC and INS, as well as self-connection of the DLPFC after rTMS treatment, with decreased connectivity from the sgACC to HIP, as well as the self-connection of the sgACC (Fig. 6c). In PCECM, depression responders showed decreased connectivity in the CAU itself following rTMS treatment (Fig. 6d).

For depression responders, the baseline (pre-treatment) self-connection of the sgACC was negatively correlated with MADRS reduction (*p* = 0.033) (Fig. 6e). The effective connectivity from the HIP to sgACC was also negatively correlated with MADRS scores after rTMS treatment (*p* = 0.040) (Fig. 6f).

## Discussion

In the current study, we examined the feasibility and clinical efficacy of SAINT in the relief of suicidal ideation in patients with MDD. Our results showed that SAINT rapidly reduced the severity of suicidal ideation with a high response rate of up to 65.63% with only 5 days of treatment. Moreover, stimulation of the DLPFC targets induced changes in a brain network of regions that had negative functional connectivity (i.e., correlations) with the target region. In addition, by comparing responders and non-responders, we found that distinct changes in connectivity may contribute to the rapid effects of SAINT on the relief of suicidal ideation and amelioration of depression severity, respectively. These findings suggest that SAINT has great promise for the treatment of suicidal ideation associated with depression. More importantly, the current study could also extend our understanding of the neurobiological underpinnings of SAINT, which could further facilitate optimization of its clinical efficacy.

The current study demonstrated that SAINT is a safe and feasible way that could rapidly and effectively alleviate suicidal ideation in patients with MDD. High suicide risk in MDD is a serious public health issue, yet an effective treatment strategy which can rapidly and safely relieve suicidality in these patients remains elusive. Currently, available treatment options such as antidepressants, lithium, and psychotherapy have failed to show rapid and effective prevention effects, whereas some may even increase suicidal thoughts in early-phase pharmacotherapy [14]. There is a growing interest in the use of rTMS to reduce suicidal ideation. However, studies have shown inconsistent benefits of rTMS on suicidal ideation [53]. Earlier sham-controlled rTMS studies have reported a reduction in suicidal ideation, but the improvements were independent of active or sham stimulation [54–56]. After the stimulation protocol was optimized and MRI-guided precision targeting strategy was employed, suicide prevention effects of rTMS seem to have been enhanced. Pan and colleagues [57] reported that MRI-navigated high-dose rTMS treatment significantly reduced suicidal ideation relative to sham stimulation.

Recent studies have also suggested that SAINT is an effective way to relieve depression, but its suicidal prevention effects remain unaddressed [23]. In this study, we found that SAINT could effectively alleviate suicidal ideation in MDD patients, with a high response rate of up to 65.63%. Moreover, the response rate reached 78.13% and 90.63% respectively for 2 weeks and 4 weeks after SAINT. These findings could promote the development of safe and rapid suicide prevention strategies and reduce the suicide risk in patients of MDD.

The current study identified the neural pathways that might support the rapid suicide prevention and antidepressant effects of SAINT. We studied the signal propagation pathways from the rTMS targets to other rTMS responsive regions by using effective connectivity analysis, which describes directed information flow within a brain network. It is thought that the propagation from the stimulation target (DLPFC) of rTMS may be an accurate biomarker for its clinical efficacy [58]. In this study, for the first time, we identified the pathways using effective analysis from the DLPFC target to core brain systems implicated in depression. Specifically, stimulation of the DLPFC might first inhibit the activity of the PCUN and INS, from which influences were then relayed to the sgACC, resulted in suppression of enhanced limbic activation in depressed patients. These findings provide crucial support for the hypothesis that rTMS may induce its antidepressant effects through remote normalization of hyperactivity in the sgACC and other limbic regions [35].

It is worth noting that, instead of a direct inhibitory connection from the target to the sgACC, the results suggest that the stimulation effects might first propagate to the INS which then relies on the sgACC, and other core brain regions implicated in MDD. Intriguingly, although the basic idea behind SAINT is to improve the treatment efficacy by targeting the region that is most negatively functionally connected with sgACC [22, 23], we did not find any significant correlations between the DLPFC-sgACC connectivity and depression score reductions in the current study. According to recent studies, the proximity (distance) between the actual target and the optimal DLPFC target was anticorrelated with the SGC-based functional connectivity strengths [29, 59]. Here, the optimal potential stimulation target has already been selected as the spot with the highest anticorrelation with the sgACC, and the actual stimulation target and the optimal stimulation target should be 0 mm, which may be one of the reasons for no significant correlation were witnessed between DLPFC-sgACC functional connectivity and depression score changes.

Indeed, it is the INS acts as a hub node in the network. This is in line with the functional anatomy of the insula and sgACC. Anatomically, previous studies have reported the absence of direct anatomical connections between BA46 (DLPFC) and BA25 (sgACC) [60]. In contrast, tracer studies and in vivo fiber tracking studies have consistently identified structural connectivity of the INS with frontal, temporal, and limbic regions in the macaque monkey and human brains [61–64]. Functionally, the INS has been considered to be a crucial functional hub [65]. It is involved in a wide range of function including emotion regulation, salience detection, attentional control, etc. [65]. More importantly, the INS was thought to initial the switching between large-scale task negative and task positive networks [66–68].

Our findings also suggest that different neural mechanisms may contribute to the rapid-acting effects of SAINT on relief of suicidal ideation and amelioration of depression severity, respectively. By comparing the effective connectivity of responders and non-responders, we found that relief of suicidal ideation was specifically associated with effective connectivity of the INS and HIP, while mitigation in the severity of depression was related to connectivity of the sgACC. Consistent evidence have related the antidepressant effects of rTMS with connectivity of the sgACC [27, 28, 69–71]. An earlier study found that better treatment outcomes were associated with more negative functional connectivity between the target and sgACC [27]. This finding was further replicated in research from other groups [28, 69, 71]. This region thus was suggested as a possible neurobiological marker for the assessment of the clinical efficacy of antidepressant treatments [71]. Baseline sgACC metabolic activity and connectivity were found to be predictable of anti-depressive response [28, 69, 71], which is also replicated in our results.

Resting-state DLPFC-sgACC functional connectivity profiles also reliably differentiated responders and non-responders [35]. Furthermore, the depression circuit maps of those responders in our current study were to some extent similar by visually inspection with the convergent network proposed by Siddiqi, Schaper [48] (Supplementary Figure 1). On the other hand, the differences between the connectivity maps of our current study and the convergent network from Siddiqi et al. [48] were may attributed to the nature of the samples included in the manuscript were MDD patients with suicidal ideation, which in agree with our finding that different neural mechanisms may contribute to the rapid-acting effects on suicidal ideation and amelioration of depression severity.

Regarding the neural pathways contributing to the suicide prevention effects of SAINT, the current study extended previous studies by showing that the effective connectivity of the INS and HIP predicts the rapid-acting effects of SAINT on suicidal ideation. The INS is one of the core regions in the brain’s salience network, which is crucial for cognitive control [36]. Among individuals with borderline personality disorder, a disorder defined partially by recurrent suicidal behavior, the suicide attempters demonstrated decreased grey matter concentrations in the INS compared with healthy controls and non-attempters [72]. Reduced cortical thickness in INS was also reported in depressed patients with suicidal ideation [73]. A recent MEG study reported reduced gamma power which reflected imbalance in excitation-inhibition in the INS in MDD patients [74].In the current study, we showed that the self-connection which is reflective of the excitatory of this INS was reduced by SAINT. Our findings suggest that SAINT may rapidly alleviate suicidal ideation through modulating the excitatory of the INS. More importantly, it may also be possible to optimize the clinical efficacy of SAINT for suicide prevention by selecting a stimulation target which demonstrates the most negative functional/effective connectivity with INS.

We need to consider some limitations when interpreting our results. This study aimed to explore the feasibility of the Stanford Accelerated Intelligent Neuromodulation Therapy (SAINT) in rapidly relieving suicidal ideation with an open-label design without sham groups according to the original SAINT study [23], we could not rule out possible confounding effects from drugs. A double-blind, randomized, sham-controlled trial is required for further investigation to better interpreting the therapy’s underlying mechanisms and benefiting it in alleviating suicide ideation and depression. In addition, a real-time target tracking and following robot system was used to ensure that the DLPFC subregion—which was most negatively functionally connected with sgACC—received the stimulation. Thus, we were unable to collecting fMRI data simultaneously when the patients were receiving rTMS stimulation, due to the difficulty of placing the robot system in an MRI scanner. Future studies may need to replicate the findings with concurrent TMS-fMRI or TMS-EEG. Another limitation is that the multiple corrections were not performed on the correlations between connectivity and clinical score because small sample size was used in this study. This study is for the first time to explore the underlying mechanism of SAINT, we should be cautious in interpreting these results and studies with large sample sizes are better to be conducted for further exploring the neural mechanism of the SAINT. Moreover, the limitation of the effectivity should be addressed here, DCM is constructed based on Bayesian model comparison or reduction, which depends upon data itself. This procedure would simplify model itself with sacrificing data complexity [75]. In addition, considering safety issue for all patients, rTMS treatment were combined with antidepressants (i.e., Venlafaxine/Duloxetine) as previous studies [22, 23, 37]. Another limitation is that the recruited patients are not persons with treatment-refractory depression, the results could not be generalized to this population group.

### Supplementary information

Supplementary materials

## Data Availability

Data supporting the findings of this study are available from the corresponding author.
