# Supplementary material for: Targeting suicidal ideation in major depressive disorder with MRI-navigated Stanford accelerated intelligent neuromodulation therapy
Source: Transl Psychiatry. 2024 Jan 10;14:21. doi: 10.1038/s41398-023-02707-9 (PMC10781692; doi:10.1038/s41398-023-02707-9)
Supplement: Supplementary file 1 — Supplementary materials [file 41398_2023_2707_MOESM1_ESM.docx]

# Supplementary Materials:

**The definition of stimulation targets**

According to previous studies, the stimulation target was defined (1, 2). Specifically, the time series of each voxel within the DLPFC and sgACC were extracted based on the individual space. Then, hierarchical clustering was used to divide these two regions into different subunits based on Spearman correlation (*r* >= 0.5) among voxels within these two regions separately. Subsequently, the Spearman correlation coefficient was calculated for each subunit, and then, the sum of all the correlations weighted by the sizes of all the bilateral sgACC subunits was conducted, i.e., net r-value. This value combined with the cluster size (voxel number) and spatial concentration of the left DLPFC, were taking into consideration for optimal subunit. Specifically, spatial concentration was calculated to divide the average Euclidean distances of each pair of voxels within this subunit by the voxel numbers of the subunit. Finally, the net r value, the DLPFC subunit size, and spatial concentration were integrated to determine the stimulation target using decision-making algorithm. As a part of the Brodmann Area 25 (BA 25) was used in Stanford Neuromodulation Therapy (1, 2) located on the corpus callosum, the No. 187 and 188 atlases based on Brainnetome Atlas (BNA) (3) (<https://atlas.brainnetome.org/bnatlas.html>) were instead selected as the sgACC in our current study.

**Safety**

No severe adverse events occurred during the trial and the most common side effect was headache (see Table 3 of supplementary materials). Other reported discomforts included scalp numbness, weeping, nausea, jaw twitching, etc. These side effects were mild, well tolerated, and resolved rapidly after stimulation. No participants dropped out due to intolerable adverse events. Neurocognitive assessments showed no cognitive side effects after SAINT. Instead, subjects’ performances were significantly improved on DST scores (*T* = − 6.155，*p* < 0.001) and DSST scores (*T* = − 6.550，*p* < 0.001). There was also a significant reduction in PDQ-D scores (*T* = 6.054，*p* < 0.001) from baseline.

Table 1. Demographic characteristics of the patients

|  | Mean | SD |
| --- | --- | --- |
| Age（years） | 27.66 | 20.38 |
| Years of education | 12.88 | 2.79 |
| Age at onset of depression | 25.75 | 9.51 |
| Dosage of medication at 2 weeks after SNT (mg/day) |  |  |
| Venlafaxine (N=28) | 150 | 0 |
| Duloxetine(N=4) | 60 | 0 |
| Dosage of medication at 4 weeks after SNT (mg/day) |  |  |
| Venlafaxine (N=28) | 196.15 | 42.83 |
| Duloxetine(N=4) | 60.00 | 0 |

Table 2. ROIs included in PCECM and NCECM analysis

| ROIs | The corresponding BNA No. |
| --- | --- |
| mPFC | 11,12 |
| CAU_L | 219 |
| HIP_L | 215 |
| PCUN_L | 151,153 |
| INS_L | 171 |
| sgACC_B | 187,188 |

Note: PCECM: positive correlation effective connectivity model; NCECM: negative correlation effective connectivity model; BNA: Brainnetome Atlas (3) (https://atlas.brainnetome.org/); mPFC: the medial prefrontal cortex; CAU_L: the left caudate; HIP_L: the left hippocampus; PCUN_L: the left precuneus; INS_L: the left insular; sgACC_B: the bilateral subgenual anterior cingulate.

Table 3. Side effect occurred during 5 days of SAINT

| **Side effect** | **N** | Proportion |
| --- | --- | --- |
| headache at the stimulating site | 18 | 56.25% |
| scalp numbness | 9 | 26.47% |
| weep | 4 | 11.76% |
| nausea | 4 | 11.76% |
| jaw twitching | 3 | 8.82% |
| discomfort in the teeth | 2 | 5.88% |
| tinnitus | 1 | 2.94% |
| palpitate | 1 | 2.94% |


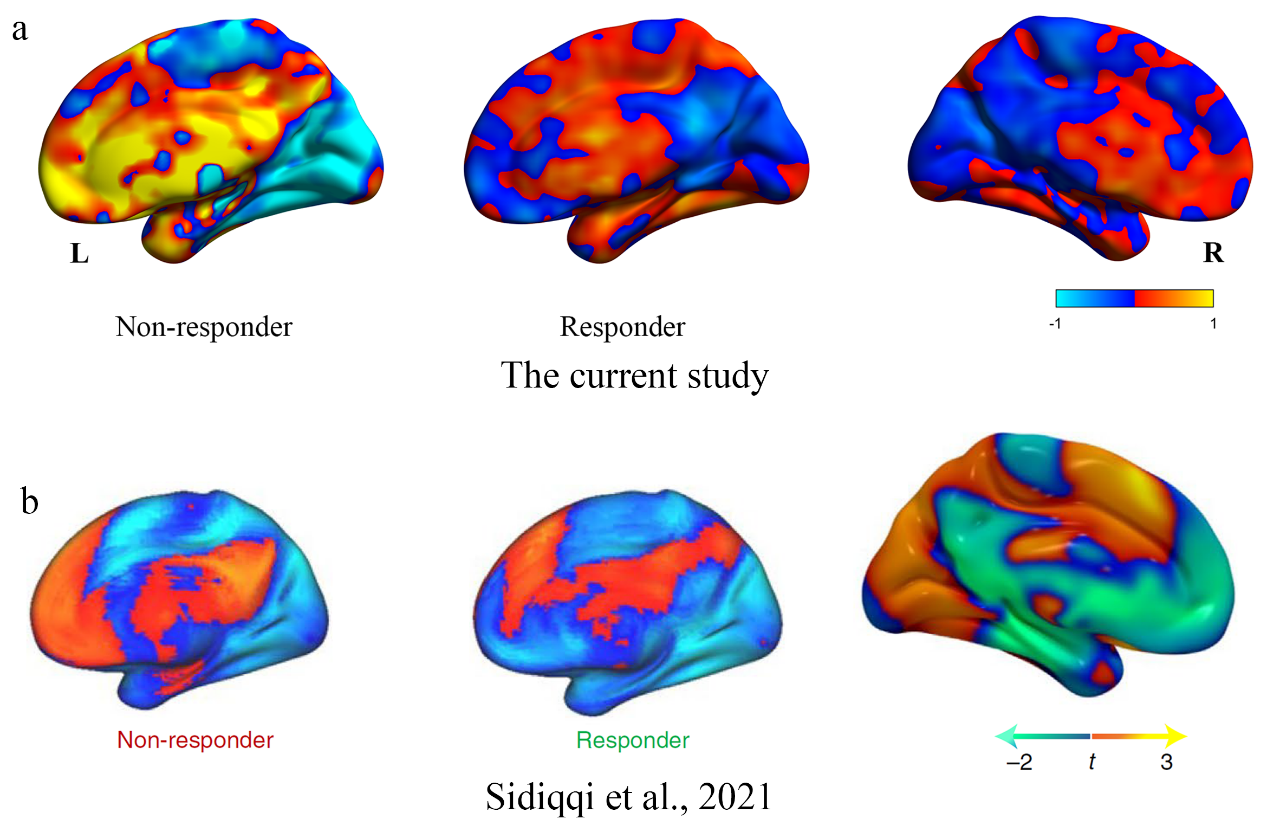


Figure 1. The depression responder and non-responder circuit maps identified according to Siddiqi, Schaper (4). a, the depression circuits identified in the current study; b, the convergent network from Sidiqqi et al., 2021; in which, similar pattern to some extent between those responders in our study and the convergent network derived from Siddiqi, Schaper (4) by inspection visualization; greater connectivity represents more decreased depression scores.

**Reference**

1. Cole EJ, Phillips AL, Bentzley BS, Stimpson KH, Nejad R, Barmak F, Veerapal C, Khan N, Cherian K, Felber E, Brown R, Choi E, King S, Pankow H, Bishop JH, Azeez A, Coetzee J, Rapier R, Odenwald N, Carreon D, Hawkins J, Chang M, Keller J, Raj K, DeBattista C, Jo B, Espil FM, Schatzberg AF, Sudheimer KD, Williams NR. Stanford Neuromodulation Therapy (SNT): A Double-Blind Randomized Controlled Trial. Am J Psychiatry. 2021:appiajp202120101429.

2. Cole EJ, Stimpson KH, Bentzley BS, Gulser M, Cherian K, Tischler C, Nejad R, Pankow H, Choi E, Aaron H, Espil FM, Pannu J, Xiao X, Duvio D, Solvason HB, Hawkins J, Guerra A, Jo B, Raj KS, Phillips AL, Barmak F, Bishop JH, Coetzee JP, DeBattista C, Keller J, Schatzberg AF, Sudheimer KD, Williams NR. Stanford Accelerated Intelligent Neuromodulation Therapy for Treatment-Resistant Depression. Am J Psychiatry. 2020;177:716-726.

3. Fan L, Li H, Zhuo J, Zhang Y, Wang J, Chen L, Yang Z, Chu C, Xie S, Laird AR, Fox PT, Eickhoff SB, Yu C, Jiang T. The Human Brainnetome Atlas: A New Brain Atlas Based on Connectional Architecture. Cereb Cortex. 2016;26:3508-3526.

4. Siddiqi SH, Schaper F, Horn A, Hsu J, Padmanabhan JL, Brodtmann A, Cash RFH, Corbetta M, Choi KS, Dougherty DD, Egorova N, Fitzgerald PB, George MS, Gozzi SA, Irmen F, Kuhn AA, Johnson KA, Naidech AM, Pascual-Leone A, Phan TG, Rouhl RPW, Taylor SF, Voss JL, Zalesky A, Grafman JH, Mayberg HS, Fox MD. Brain stimulation and brain lesions converge on common causal circuits in neuropsychiatric disease. Nature human behaviour. 2021;5:1707-1716.
